# PPAR Gamma Activity and Control of Bone Mass in Skeletal Unloading

**DOI:** 10.1155/PPAR/2006/64807

**Published:** 2006-09-04

**Authors:** P. J. Marie, K. Kaabeche

**Affiliations:** ^1^Laboratory of Osteoblast Biology and Pathology, INSERM U606, 75475 Paris Cedex 10, France; ^2^Faculty of Medicine, University of Paris 7, 75251 Paris Cedex 05, France

## Abstract

Bone loss occuring with unloading is associated with decreased osteoblastogenesis and increased bone marrow adipogenesis, resulting in bone loss and decreased bone formation. Here, we review the present knowledge on the role of PPARγ in the control of osteoblastogenesis and bone mass in skeletal unloading. We showed that PPARγ positively promotes adipogenesis and negatively regulates osteoblast differentiation of bone marrow stromal cells in unloading, resulting in bone loss. Manipulation of PPARγ2 expression by exogenous TGF-β2 inhibits the exaggerated adipogenesis and corrects the balance between osteoblastogenesis and adipogenesis induced by unloading, leading to prevention of bone loss. This shows that PPARγ plays an important role in the control of bone mass in unloaded bone. Moreover, this opens the possibility that manipulation of PPARγ may correct the balance between osteoblastogenesis and adipogenesis and prevent bone loss, which may have potential implications in the treatment of bone loss in clinical conditions.

## INTRODUCTION

The maintainance of both bone mass and bone microarchitecture is controlled by the balance between bone resorption and formation. At the cellular level, this balance is largely dependent on the number and activity of bone forming and resorbing cells. Any alteration in the number or activity of bone cells will result in an imbalance between resorption and formation, resulting in microarchitecture deterioration and altered bone mass and strength.

The control of bone forming cells is largely influenced by weight bearing and exercise that induce mechanical forces on the skeleton. Mechanical forces induce anabolic effects by promoting bone formation at multiple levels [1–3]. Bone formation is a complex process that is dependent on the recruitment, differentiation, and function of osteoblasts. The osteogenic process starts by the commitment of osteoprogenitor cells into osteoblasts under the control of transcription factors, followed by their progressive differentiation into mature osteoblasts [4, 5]. In the recent years, the development of cellular, molecular, and genetic studies has led to the identification of a number of important transcription factors that are essential in the control of bone formation. Specifically, several studies have provided evidence for a role of PPARγ in the control of bone formation and bone mass through modulation of bone marrow stromal cell differentiation. In this brief review, we summarize the present knowledge on the role of PPARγ in the control of osteoblastogenesis and bone mass, with a particular reference to skeletal unloading.

### Reciprocal relationship between osteoblastogenesis and adipogenesis in the bone marrow

Several conditions associated with bone loss such as aging [6], glucocorticoid treatment [7], estrogen deficiency [8], or immobilization [9] are characterized by decreased osteoblastogenesis associated with increased adipogenesis in the bone marrow. This supports the concept that there is a reciprocal relationship between adipocyte and osteoblast differentiation [10]. Early studies showed that bone marrow stromal cells can be differentiated into several lineages in vitro [11–13], and that differentiation towards one lineage is dependent on local or hormonal factors [14]. Further studies showed that clonal marrow stromal cells can be differentiated into adipocytes, osteoblasts, or chondrocytes in different species including humans [15–17]. Notably, a single marrow stromal cell may have multipotential competence in vitro and differentiation towards one pathway restricts expression of other lineage-specific genes [18]. This provides evidence that adipocytes and osteoblasts are derived from a common mesenchymal stromal cell and that a reciprocal relationship exists between osteoblastogenesis and adipogenesis in the bone marrow [10].

### PPARγ2 is a positive promoter of adipogenesis and a negative regulator of osteoblastogenesis

The mechanisms involved in adipogenesis have been studied extensively in adipose tissue. The differentiation of preadipocytes into mature adipocytes is primarily controlled by peroxisome proliferator-activated receptor γ (PPARγ) which is a key transcription factor involved in adipocyte differentiation [19]. PPARγ exists in two isoforms PPARγ1 and PPARγ2 as a result of alternative splicing. PPARγ2 is expressed at high levels in fat tissue and is essential for adipogenesis in vitro and in vivo. CCAAT/enhancer binding proteins (C/EBP) are other important transcription factors that control the expression of adipocyte genes by acting synergistically with PPARγ to activate adipocyte gene expression [20]. In vitro, C/EBPs activate the expression of PPARγ and C/EBPα and promote PPARγ2 activity in preadipocyte cultures, which contributes to the expression of genes that characterize the adipocyte phenotype [21].

**Figure 1:**
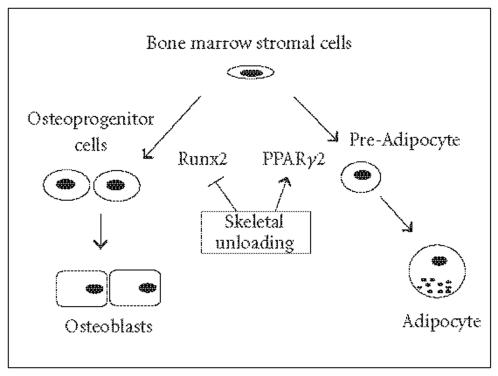
The in vivo differentiation of bone marrow stromal cells towards adipocytes and osteoblasts is governed by the balance between PPARγ2 and Runx2 expression. In unloaded bone, decreased Runx2 and increased PPARγ2 expression result in decreased osteoblastogenesis, increased adipogenesis, and bone loss.

In bone, recent advances have been made in the role of PPARγ in the interconversion of marrow stromal cells into osteoblasts or adipocytes in vitro (Figure 1). In cultured murine and human cells, PPARγ agonists and overexpression of PPARγ2 induce the differentiation of bone marrow stromal cells into the adipocyte lineage and negatively regulate osteoblast differentiation by repressing the osteoblast specific transcription factor Runx2 [22–24]. There is also evidence that PPARγ negatively regulates osteoblast differentiation. For example, activation of PPARγ with a thiazolidinediones with high affinity for PPARγ increases adipogenesis and decreases osteoblastogenesis in vitro [25–27]. Additionally, activation of PPARγ with rosiglitazone in mice or ovariectomized rats decreases Runx2 expression and bone formation, and increases adipogenesis in the bone marrow, resulting in decreased bone mass [28, 29]. Consistently, PPARγ haploinsufficiency in mice was shown to decrease adipogenesis and to increase Runx2 expression and bone formation, resulting in increased bone mass [30]. These findings indicate that PPARγ positively promotes adipogenesis and negatively regulates osteoblast differentiation of bone marrow stromal cells in vivo, suggesting that PPARγ is a negative regulator of bone mass.

### Skeletal unloading decreases osteoblast differentiation and induces bone loss

A representative model of bone loss resulting from alterations in osteoblasts is skeletal unloading [31]. Skeletal unloading induced by hind limb suspension rapidly causes a marked trabecular bone loss in the long bone metaphysis, resulting mainly from reduced trabecular thickness and number associated with inhibition of endosteal bone formation [32]. Although both the number and activity of osteoblasts are decreased in the unloaded metaphyseal bone [32, 33], the number of osteoblasts is more affected than their activity [34]. Although the mechanisms underlying bone loss induced by unloading in rats are not fully understood, bone loss does not appear to result from changes in serum corticosteroid, 25-hydroxyvitamin D or PTH levels [31]. However, there is some evidence that skeletal unloading may result in part from to decreased expression [34] or response [35] to local growth factors.

The cellular mechanisms underlying the alterations of bone formation induced by skeletal unloading in rats have been partly identified [36]. We initially showed that the decreased bone formation in unloaded rat bone results from an impaired recruitment of osteoblast precursor cells in the bone marrow stroma and in the metaphysis [33]. In addition to affect osteoblast recruitment, skeletal unloading in this model alters the function of differentiated osteoblasts. This is reflected by the decreased expression of bone matrix type-1 collagen and osteocalcin and osteopontin mRNA levels [37–40], which correlates well with the decreased bone matrix synthesis measured at the tissue level [32, 33]. These findings indicate that removal of mechanical forces on the skeleton rapidly alters both the recruitment of osteoblast progenitor cell and the function of differentiated osteoblasts, resulting in a marked reduction of bone formation. Such alterations are consistent with the effects of unloading in other rat models in which there is a reduction of the osteogenic capacity of bone marrow osteoblast precursor cells and a decreased expression of bone matrix proteins in rat long bones [41, 42].

### PPARγ controls the osteoblast/adipocyte relationship in unloaded bone

The altered bone metabolism induced by skeletal unloading is asociated with alterations in transcription factor expression. Specifically, the decreased osteoblastogenesis and bone formation induced by skeletal unloading in rats are associated with reduced Runx2 expression [34]. Additionally, we showed that skeletal unloading is associated with increased adipocyte differentiation in the bone marrow stroma [43], suggesting that unloading not only impairs osteoprogenitor cell differentiation into osteoblasts but also promotes adipocyte differentiation. The exagerated reciprocal relationship between osteoblastogenesis and adipogenesis may account for the decreased bone formation associated with the increased bone marrow adipogenesis in unloaded rats (Figure 1).

**Figure 2:**
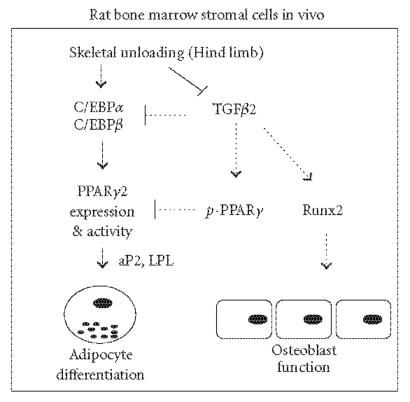
Skeletal unloading decreases TGF-β expression and activates the expression of C/EBPα, C/EBPβ, and PPARγ2, resulting in activation of adipocyte gene expression such as adipocytic differentiation-related genes adipocyte binding protein (aP2) and lipoprotein lipase (LPL) in bone marrow stromal cells. Exogenous TGF-β2 (dotted lines) reduces C/EBPα, C/EBPβ, and PPARγ expressions, induces PPARγ phosphorylation (*p*-PPARγ), and increases Runx2 expression, resulting in decreased adipogenesis, increased osteoblast function, and prevention of bone loss.

Interestingly, the adipogenic differentiation of bone marrow stromal cells in unloaded bone is consistent with the temporal gene expression observed during adipocyte differentiation in vitro. Specifically, skeletal unloading in rats increases C/EBPα and C/EBPβ expression followed by increased expression of PPARγ, resulting in activation of adipocyte gene expression such as adipocytic differentiation-related genes adipocyte binding protein (aP2) and lipoprotein lipase (LPL) in bone marrow stromal cells [44] (Figure 2). Thus, PPARγ with other transcription factors are involved in adipogenic conversion of bone marrow stromal cells in vivo, indicating that PPARγ is a negative regulator of bone mass in unloaded rats.

The mechanisms underlying the expression of Runx2 and PPARγ in unloaded bone may involve decreased signaling pathways that are normally transmitted by loading. Mechanical forces are believed to transduce signals through cell-matrix interactions [45–48]. Part of the communication between the matrix and cells is ensured by integrins which interact with bone matrix proteins [49]. In bone, integrin-matrix interactions are important modulators of osteoblast differentiation in vitro [50, 51]. It is thus possible that the lack of mechanical strain is induced by unloading results in decreased integrin-matrix interactions and signaling, and consequently decreased osteoblast differentiation. This is supported by the finding that mechanical forces increase Runx2 expression in cultured preosteoblastic cells [52]. One recent study indicates that stretching induces downregulation of PPARγ2 and adipocyte differentiation in mouse preadipocytes [53], suggesting that mechanical forces may play a dual role in the control of Runx2 and PPARγ expression in preosteoblasts.

How mechanical signals may modulate PPARγ expression or activity and thereby induce adipogenesis rather than osteoblastogenesis in bone marrow stromal cells is not fully understood. One interesting hypothesis is that specific pathways controlling osteoblastogenesis/adipogenesis may be sensitive to biomechanical forces. For example, changes in cell shape or modulation of the cytoskeletal-related GTPase RhoA were recently found to induce stem cell adipogenic or osteoblast differentiation [54]. Additionally, multiple signal pathways, including ERK and Wnt signaling, may control the balance between adipogenesis and osteoblastogenesis in vitro [53, 55]. It remains however to determine which pathway may be involved in the altered balance between osteoblastogenesis and adipogenesis in vivo.

### TGF beta is a negative regulator of PPARγ and adipogenesis in unloaded rats

Transforming growth factor beta (TGF-β) is an important regulator of bone formation by modulating osteoblastic cell proliferation and differentiation [56]. Additionally, TGF-β is also an important modulator of adipocyte differentiation. TGF-β inhibits adipogenesis in preadipocyte cell lines and reduces adipocyte differentiation in vitro [57, 58]. In vivo, we found that skeletal unloading results in a rapid reduction in TGF-β1 and TGF-β receptor II mRNA expression in bone marrow stromal cells [34]. Others found reduced TGF-β2 mRNA levels in bone marrow stromal cells in this model [37], suggesting that TGF-β signaling may mediate part of the altered bone formation induced by unloading. Although diminished, TGF-β receptors can still be activated by TGF-β since we showed that exogenous TGF-β2 in unloaded rats increased Runx2 expression and osteoblastogenesis, resulting in prevention of trabecular bone loss [59]. Beside this positive effect on osteoblastogenesis, TGF-β2 administration downregulated the expression of C/EBPα, C/EBPβ, and PPARγ in bone marrow stromal cells, and reduced the expression of adipocyte genes such as aP2 and LPL in bone marrow stromal cells, thus preventing the adipocyte conversion of bone marrow stromal cells induced by unloading [43, 44]. This indicates that TGF-β is a negative regulator of PPARγ and adipogenesis in unloaded rats (Figure 2).

One mechanism by which TGF-β may negatively regulate adipogenesis in unloaded rats is through MAPK activation. TGF-β is known to induce phosphorylation of PPARγ in adipocyte cells, and MAPK-dependent PPARγ phosphorylation results in the reduction of PPARγ transcriptional activity and repression of adipocyte differentiation [60–62]. In vitro, ERK activation was found to induce osteogenic differentiation of human mesenchymal stem cells, whereas its inhibition induces adipogenic differentiation [63]. In unloaded bone, we showed that TGF-β2 increased PPARγ phosphorylation and inhibited adipocyte differentiation of bone marrow stromal cells through MAPK phosphorylation [44]. Thus, exogenous TGF-β can inhibit the excessive adipogenic differentiation induced by skeletal unloading by reducing PPARγ2 expression, resulting in the inhibition of adipogenesis. This effect, combined with the upregulation of Runx2 expression and osteoblast differentiation induced by exogenous TGF-β on bone marrow stromal cells, leads to correcting the imbalance between osteoblastogenesis and adipogenesis and results in a positive effect on bone mass (Figure 2). This demonstrates that appropriate manipulation of PPARγ2 expression in vivo can lead to prevent bone loss in unloaded bone.

## CONCLUSION

There is now clear evidence that PPARγ plays an important role in the control of marrow stromal cell differentiation to osteoblasts or adipocytes in unloaded bone. In this model, PPARγ positively promotes adipogenesis and negatively regulates osteoblast differentiation of bone marrow stromal cells, indicating that PPARγ is a negative regulator of bone mass. This concept provides a possible target for therapeutic intervention in osteopenic disorders characterized by altered osteoblast and adipocyte differentiation of bone marrow stromal cells [64]. As an example, we showed that exogenous manipulation of PPARγ expression by TGF-β can inhibit adipogenesis induced by skeletal unloading and correct the balance between osteoblastogenesis and adipogenesis, resulting in prevention of bone loss. This opens the possibility that manipulation of PPARγ may have potential implications in the treatment of bone loss associated with immobilization [65].
